# QTL detection for a medium density SNP panel: comparison of different LD and LA methods

**DOI:** 10.1186/1753-6561-4-s1-s10

**Published:** 2010-03-31

**Authors:** Olivier Demeure, Nicola Bacciu, Olivier Filangi, Pascale Le Roy

**Affiliations:** 1 INRA, UMR 598 Génétique Animale, F-35000 Rennes, France; 2 Agrocampus-Ouest, UMR Génétique Animale, F-35000 Rennes, France

## Abstract

**Background:**

New molecular technologies allow high throughput genotyping for QTL mapping with dense genetic maps. Therefore, the interest of linkage analysis models against linkage disequilibrium could be questioned. As these two strategies are very sensitive to marker density, experimental design structures, linkage disequilibrium extent and QTL effect, we propose to investigate these parameters effects on QTL detection.

**Methods:**

The XIIIth QTLMAS workshop simulated dataset was analysed using three linkage disequilibrium models and a linkage analysis model. Interval mapping, multivariate and interaction between QTL analyses were performed using QTLMAP.

**Results:**

The linkage analysis models identified 13 QTL, from which 10 mapped close of the 18 which were simulated and three other positions being falsely mapped as containing a QTL. Most of the QTLs identified by interval mapping analysis are not clearly detected by any linkage disequilibrium model. In addition, QTL effects are evolving during the time which was not observed using the linkage disequilibrium models.

**Conclusions:**

Our results show that for such a marker density the interval mapping strategy is still better than using the linkage disequilibrium only. While the experimental design structure gives a lot of power to both approaches, the marker density and informativity clearly affect linkage disequilibrium efficiency for QTL detection.

## Background

New molecular technologies, like DNA sequencing and SNP detection, allow high throughput genotyping for QTL mapping with dense genetic maps. Thus, the classical linkage analysis (LA) methods can be improved by the integration of linkage disequilibrium information (LDLA) or considered useless, in benefits of LD methods. However, the relative interest of the two approaches depends on several parameters, like the experimental design (number and size of families), the LD status between QTL and markers, the density of genetic map, the QTL effects on traits and so on. In this study, we investigate some of these points, using the XIIIth QTLMAS workshop simulated dataset.

## Methods

### Simulated data

Data for 2.025 individuals across 2 generations were simulated [1]. In the first generation 5 sires were mated with 20 dams for giving 2000 offspring divided into 100 full sib families which are coming form all possible sire-dam combinations (20 offspring per family). All individuals are genotyped for 453 SNP markers distributed over 5 linkage groups and only individuals coming from 50 families were phenotyped for one trait measured at 5 different time points across the production curve.

### Models

Data were analyzed by fitting several models and results compared to each other.

In three first models, the markers are assumed to only affect the trait if they are in linkage disequilibrium with a QTL (LD models).

The first model was fitted by not taking into account any population structure. The association between the marker and the trait was tested using a marker fixed effect with 4 levels (00, 01, 10, 11):

**y = μ + Xg + e	  (LD1)**

Dimensions: (4)

Where** y** is a vector of phenotypes,** X** is a design matrix allocating records to the marker effect,** g** is the effect of the marker and** e** is a vector of random deviates ~* N* (0,** σ_e_^2^**), where **σ_e_^2^** is the error variance.

The second model considers the association between the marker and the trait while taking into account also the parental effect:

**y = µ + sire + dam + Xg + e   (LD2)**

Dimensions: (5)  (20)  (4)

Where dam is the dam fixed effect, sire is the sire fixed effect. 

The third model considers the SNP alleles effect:

**y = µ + sire + dam + HS + HD + e   (LD3)**

Dimensions:  (5)   (20)  (2)  (2)

Where HS and HD are the marker alleles received by one progeny from the sire and from the dam, respectively.

The last model is a linkage analysis model taking into account the parental haplotype received by a progeny from its parent, within family:

**y = µ + sire + dam + HS (sire) + HD (dam) + e   (LA)**

### Statistical methods

The 4 linear models were applied marker by marker using the SAS GLM procedure [2]. The association between the marker and the trait was tested, marker by marker, by the significance of the marker or haplotype fixed effect; eventually by parental sex. As the LA analyses were also performed marker by marker, it will be further mentioned as MLA.

The LA model was also applied in a QTL interval mapping way (further called IMLA), using the QTLMAP software [3] which was developed for populations containing a mixture of full and half-sib families [4]. The presence of the QTL was assessed using the ratio of likelihood under the hypothesis of one vs. no QTL linked to a given set of markers [5]. A fast algorithm was developed to estimated transmission probabilities at each location of a linkage group according to the SNPs information [6]. QTLMAP software was also used to test some more complex hypotheses, like two linked QTLs influencing the same trait [7]. In this case, the H1 hypothesis (there is one QTL on the linkage group) is compared to the H2 hypothesis (there are two QTLs in the linkage group). The two QTL locations under H2 are estimated considering all possible combinations based on a two dimension grid. This is of particular interest to test if a QTL detected in a single QTL LA could be a ghost.

Finally, a possible interaction between QTLs was evaluated by using one QTL previously detected as a fixed effect and testing a possible interaction between this QTL and the rest of the linkage group. For each progeny, the level of the fixed effect is deduced according to the probability of allele transmission at the QTL location if this probability is higher than 0.8 or lower than 0.2, and other progeny are discarded. Doing this, the effect of this known QTL should be suppressed and a possible other QTL could be detected. In this case, the test only considers previously observed QTL against other locations and only within a linkage group. Two interacting QTLs without main effects or on different linkage groups cannot be detected.

For all these analyses, significance thresholds were determine by simulating the performances assuming a polygenic model with a given heritability (h2=0.5). For the two QTL model, the most likely location and effect estimated under the single QTL hypothesis are used to add this QTL effect to the performances. Up to 200 simulations were performed for each trait** x** linkage group and thresholds of rejection were estimated according to Harrel and Davis method [8].

## Results and discussion

### Linkage disequilibrium analyses

**Figure 1:**
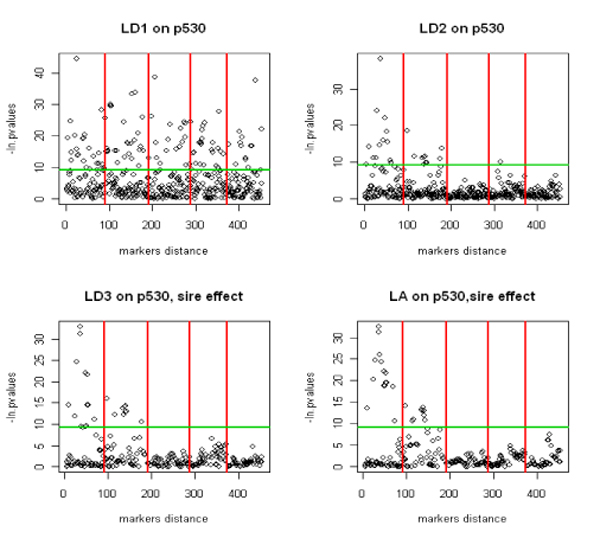
Single QTL detection with the LD and LA models for P530 (P<0.0001). Each SNP p- value is plotted based on its physical location. The linkage groups are separated by red lines. For LD1 and LD2, the overall model effect is used while the sire effect is used for LD3 and LA models. The threshold line corresponds to the natural logarithm of 0.0001 and is common to all models.

The first model identifies a very large number of significant SNPs across all the linkage groups (Figure 1). When the polygenic effect was introduced in the model we were able to identify a smaller number of significant SNPs across linkage groups which can give a better idea of the association between marker and QTL. The third model (LD3) gave results similar to those obtained by LD2.

### Single QTL linkage analysis

Single QTL interval mapping analysis results in the detection of 9 additive QTLs, summarized in Table 1. Interestingly, some of the QTLs are detected only at some time points, revealing that the trait genetic determinism evolves during the time. Most of the identified regions were not identified by the MLA. In addition, except for the first chromosome, the MLA analysis does not gives a precise location for the QTL, and it did not detected the evolution of the QTL effects during the time.

**Table 1:** Locations, effects and test statistic values for the QTLs detected by the different analyses.

		Trait P0		Trait P132		Trait P265		Trait P397		Trait P530	
		
Chr	Loc.	LRT	Effect	LRT	Effect	LRT	Effect	LRT	Effect	LRT	Effect
1	43.62	155.0****	14	174.4****	16	193.0****	18	203.2****	18	211.0****	17
2	3.7									71 2***	8
2	42.7	74 9***	9	77.0***	8	76.0***	10	70.8***	9		
3	1.28	7.1 ns		7.8 ns		7.7 ns		7.8 ns		7.3 ns	
3	17.3	58.6**	7								
3	48.7					15.5**		17.3**		15.7**	
3	92.3			54.87*	7	56.3*	7	55.6*	5	57.0*	10
4	9.3	76.5****		55.9****							
4	65.3					59.25*	7	48.45*	8	45.7*	8
4	75.28	80.7****	5	70.6***	6						
5	72.1							61.4**	10	64.3**	8
5	80.0			17.5**		18.6**		18.4**		16.5**	
5	94.1	56.2*	13	58.7*	13	60.2**	11				

Loc. = QTL location (in cM)

LRT = likelihood ratio test value

Eff. = percentage of phenotypic variance explained

*: P < 0.05, **: P < 0.01, ***: P < 0.001, ****: P < 0.0001

**Figure 2:**
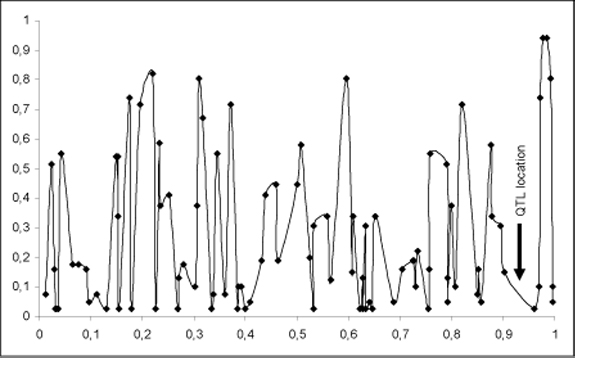
Chromosome 3 markers informativity in the population. The informativity values correspond to the average of transmission probabilities at the SNP location. The arrow highlight the location of the QTL detected at 92cM.

**Figure 3:**
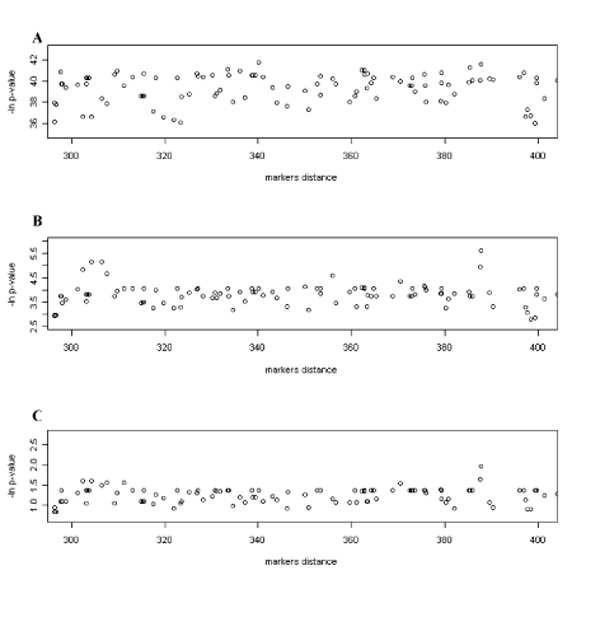
MLA results on chromosome 3 with all parents (A), selected sires (B) and selected sires and dams (C)

These discrepancies between the MLA and IMLA analyses could be mostly due to two parameters. The first one is the marker density (about one SNP every cM) and informativity. Even in regions with low informativity, the interval mapping method can calculate a probability of allele transmission by using flanking markers, while single point analysis will loose all its power. This is particularly striking for the distal region of chromosome 3 (92cM). At this location, QTLMAP detects a significant QTL (P<0.05), while MLA analysis does not. The very low informativity of the markers in this region (see Figure 2) probably explains these observations. This suggests that we would get similar results using point by point or multipoint approaches with a very dense genetic map and/or large QTL effects. The second point is the test statistics used for the QTL detection. The MLA method performs a Fisher test at the marker location while QTLMAP performs a likelihood ratio test, estimating both the QTL effect and location. Performing a MLA on sires selected based on their heterozygozity for a QTL or on subsets of families (selection of sires and dams), the QTL on chromosome 3 can be detected (see Figure 3).

### MultiQTL analysis

The two-QTLs vs. one-QTL tests were performed on all chromosomes and traits. With this model, two additive QTLs can be identified (either their effects are reciprocal or not), but there is no interaction testing. Two QTLs (chromosome 4, at 9.3 and 75.3cM) were identified as having a significant effect on the trait at P0 and P132.

When using the QTL observed on chromosome 3 at 17cM as a fixed effect, a QTL was detected at 48.7cM.

Finally, testing interaction between previously detected QTL and other locations on its linkage group, two new QTL were identified: one non significant on chromosome 3, at 1.3cM and interacting with the QTL previously identified at 17cM and another one on the chromosome 5, at 80cM and interacting with the QTL identified at 72cM. For both regions, neither the single QTL analysis nor the multiQTL analysis did identified QTLs, for any trait. Another interesting result is that for the second linkage group, at P0, a highly significant QTL is observed at 0.7cM when testing interaction with the QTL located at 43cM, while when doing a single QTL analysis, this QTL has no effect on the trait until P263.

### Comparison of LD and LA methods

As illustrated in Figure 1, the different LD models detect QTL only on chromosomes 1, 2 and 4 but with a very low accuracy in the location. The results obtained by MLA are very similar. On the opposite, IMLA (QTLMAP) detects more QTL and can identify two QTL located on the same linkage group.

### Comparison of detected QTL with simulated QTL

As IMLA performed by QTLMAP gave the best results, only the QTL identified trough this method will be compared with the simulated QTL. Of the 13 QTL identified using all the different strategies (9 by single QTL analyses and 4 by multiple QTL analyses), five are located at less than 5cM of one of the simulated QTL (see Table 2). Five other QTL were located between 5 and 10cM of a simulated QTL location. Two detected QTL are 11 and 17cM away of the most probable location and could be considered as false positives. For the QTL on chromosome 5 (77.19cM) affecting the asymptote, we found two flanking QTL (72 and 80cM). This is probably a bias in the analysis due to the data structure. In the end, 10 of the 18 QTL were detected, 5 with a good accuracy in the location and 3 reported QTL were false positive. Effects of the detected QTL mostly overestimate the simulated QTL effects. This bias is often observed and could be due to the "additive" analysis strategy by linkage group, to the possible confusion between polygenic and QTL effects with the sire/dam model used [9] and also to the use of time point traits instead of growth curve.

**Table 2:** Comparison of the detected QTL with the simulated data.

		QTLMAS		QTLMAP					
		
Trait	Chr	Location	Effect	Location	Effect	Traits affected	Δ location
Ass	1	42.5	29.3	43.6	17	P0 to P530	1.2
Ass	2	4.6	7.1	3.7	8	P530	0.9
Ass	2	88.6	3.7	-	-	-	-
Ass	3	89.9	4.1	92.3	7	P132 to P530	2.4
Ass	4	70	3.3	65.3	8	P265 to P530	4.7
Ass	5	77.2	2.5	72.1	8	P397 to P530	5.1
Growth	1	87.7	23.4	-	-	-	-
Growth	2	48.9	4.8	42.7	9	P0 to P530	6.2
Growth	3	26.2	4.7	17.3	7	P0	8.9
Growth	4	9.6	5.9	9.3	-	P0 to P 132	0.3
Growth	4	86.4	6.6	-	-	-	-
Growth	5	31.5	4.6	-	-	-	-
Inf	1	54.3	32.3	-	-	-	-
Inf	2	33	3.5	-	-	-	-
Inf	3	6.9	3.5	1.3	-	P0 to P530	5.6
Inf	3	56.1	3.8	48.7	-	P265 to P530	7.4
Inf	4	36.5	3.2	-	-	-	-
Inf	5	59.7	3.7	-	-	-	-
FALSE	4	86.4	6.6	75.3	6	P0 to P132	11.1
FALSE	5	59.7	3.7	94.1	13	P0 to P265	16.9
FALSE	5	-	-	80	-	P132 to P530	-

Location = QTL location (in cM)

Effect = percentage of phenotypic variance explained

Traits affected = traits for which this QTL was detected by QTLMAP

QTLMAP is freely available through the Quantitative Genetic Platform (QGP) at the following address: https://qgp.jouy.inra.fr/

## Conclusion

Our results show that for such a marker density the interval mapping strategy still gives better results than using the linkage disequilibrium models only. While the experimental design structure gives a lot of power to both approaches, the marker density and informativity clearly affect linkage disequilibrium efficiency for QTL detection. Also, using an interval mapping strategy offers the possibility to test interactions between markers. However, the LDLA strategy has not been tested and should improve the QTL detection.

## Competing interests

The authors declare that they have no competing interests.

## Authors' contributions

OD, NB and OF performed the statistical analyses. PLR supervised the researches. OD, NB and PLR drafted the manuscript. All authors read and approved the final manuscript.
